# Advancements in Applications of Manufacturing and Measurement Sensors

**DOI:** 10.3390/s25020454

**Published:** 2025-01-14

**Authors:** Yiping Shao, Shichang Du, Delin Huang

**Affiliations:** 1 College of Mechanical Engineering, Zhejiang University of Technology, Hangzhou 310023, China; syp123gh@zjut.edu.cn; 2 Ningbo Yongxin Optics Co., Ltd., Ningbo 315040, China; 3 School of Mechanical Engineering, Shanghai Jiao Tong University, No. 800 Dongchuan Road, Shanghai 200240, China; 4 School of Intelligent Manufacturing and Control Engineering, Shanghai Polytechnic University, Shanghai 201209, China; dlhuang@sspu.edu.cn

Manufacturing and measurement sensors are an integral part of advanced manufacturing technology, which requires sensors that can precisely capture and analyze various physical parameters during the manufacturing process [1]. These sensors have demonstrated remarkable achievements in the field of advanced manufacturing, especially in ensuring product quality and improving production efficiency [2,3]. However, the application of these sensors in the manufacturing domain has been accompanied by challenges. One of the main challenges is the complex and variable manufacturing environment, which requires sensors to maintain high reliability, stability, and accuracy under various adverse conditions [4]. Indeed, despite their widespread use, manufacturing and measurement sensors often face difficulties in adapting to extreme temperatures, high pressures, and strong electromagnetic interferences, which may affect the accuracy of the measurement data [3]. This situation raises concerns, especially in industries where high-precision manufacturing is crucial, as accurate sensor data are essential for ensuring product quality and process control [5].

In this regard, the development of advanced sensor technologies and intelligent algorithms has become a key focus in this field. For example, the emergence of smart sensors with self-calibration, self-diagnosis, and self-adaptation functions can effectively improve the performance of sensors in complex environments [6,7,8]. In particular, these advanced sensors and algorithms are designed to improve adaptability and accuracy in sensing applications, aiming to better meet the needs of modern manufacturing processes [9]. The primary objective is to enhance the reliability and effectiveness of manufacturing and measurement sensors in complex industrial scenarios [10]. This is of the utmost importance in modern manufacturing, where such improvements can have a significant impact, reducing production costs, improving product quality, and enhancing overall competitiveness [11]. In this context, several advanced sensor-based applications have been emerging in manufacturing, with applications in intelligent manufacturing systems, quality monitoring and control, and machine vision-based inspection [12,13,14].

Furthermore, the continuous expansion of the Internet of Things (IoT) in the industrial field has led to an increase in the availability of data from interconnected sensors. This rich data source provides a solid foundation for training and optimizing sensor-related models [15]. In addition, recent advances in sensor-manufacturing materials and processes have also improved the performance of sensors, which enable them to better meet the diverse requirements of modern manufacturing [16].

In this context, this topical collection includes eleven papers focused on the latest advancements in the field of manufacturing and measurement sensors. Each of the papers underwent a rigorous review process by multiple expert reviewers across several rounds of revision. The studies published in the current topical collection cover a wide range of topics, including advanced measurement sensors, measurement and sensing applications, manufacturing systems, applications of sensors in intelligent logistics equipment, quality monitoring and control, machine vision and applications, and methods of detection. The following provides a brief summary of these studies:

In Contribution 1, Luo, L. et al. introduce a high-precision capacitive pressure sensor for TPMS. By leveraging a CMUT structure and silicon–s ilicon bonding, the sensor achieves high accuracy, ultra-low power consumption, and cost-effectiveness. Rigorous tests show that it can precisely monitor tire pressure, ensuring automotive safety and reliability, which is crucial for the automotive manufacturing industry.

In Contribution 2, Wu, B. et al. explore the roundness of small cylindrical workpieces. Using the stitching linear scan method, they compare ruby ball and diamond styluses. Their experiments reveal that the ruby ball stylus is more suitable for small needle rollers, offering practical guidance for small-scale manufacturing quality control processes.

In Contribution 3, Zhu, Y. et al. develop a dual-band binocular stereo-imaging system. Integrating infrared and visible light capabilities, it can accurately measure distances and recognize objects. This system has proven effective in complex industrial environments, making it valuable for large-scale product inspection and robotic operations in intelligent manufacturing.

In Contribution 4, Du, B. et al. propose a 2D FSM angle measurement system based on diffuse reflection. The system can achieve large-angle, linear measurements with high precision. In optical equipment manufacturing, this allows for the accurate adjustment of optical components, which is essential for ensuring the quality of optical products.

In Contribution 5, Zhu, Y. et al. present a four-step infrared image algorithm. Through image segmentation, stretching coefficient calculation, layer separation, and image fusion, it significantly improves image quality and processing speed. This algorithm is effective in detecting defects in manufacturing processes, enhancing quality monitoring.

In Contribution 6, Roos-Hoefgeest, S. et al. establish a laser profilometer simulation model. By incorporating Perlin and Gaussian noise to mimic real-world speckle interference, the model accurately represents surface geometries. It serves as a powerful tool for sensor design and evaluation, which helps to improve measurement accuracy.

In Contribution 7, Yin, Y. et al. combine GNSS and IMU data using error state Kalman filtering and smoothing. This approach effectively reduces errors and enhances localization accuracy. In manufacturing logistics and automation, it ensures the precise positioning of equipment and products, which improves operational efficiency.

In Contribution 8, Jin, S. et al. analyze RV reducer rotation errors through deep Gaussian processes. Their analysis identifies key factors affecting reducer performance. In high-precision manufacturing, this knowledge helps manufacturers improve the reliability and accuracy of RV reducers.

In Contribution 9, Shao, Y. et al. put forward a point-cloud-driven pallet pose method. This method increases pose estimation accuracy by 35% and reduces feature extraction time by 30%. In manufacturing logistics, it strengthens the stability of the system, enhancing overall logistics efficiency.

In Contribution 10, Lyashenko, I.A. et al. design a high-precision tribometer. By overcoming motor backlash and signal drift, it can accurately measure adhesive contact forces. This is crucial for optimizing bonding processes in manufacturing, especially in electronics and composite material production.

In Contribution 11, Chen, X. et al. introduce a 3D metrology method using anamorphic lenses. Based on double optical centers, this method can efficiently reconstruct 3D data. This method simplifies manufacturing inspection processes and also has applications in car navigation systems, which expands its utility to different fields.

In summary, this topical collection addresses various crucial challenges and advancements in the field of manufacturing and measurement sensors. It introduces innovative sensor designs, advanced algorithms, and novel applications, which have great potential for enhancing manufacturing efficiency and product quality. We are extremely grateful to the Managing Team of *Sensors* journal for their unwavering support during the compilation of this collection. We also sincerely thank all the contributing authors for their excellent research, as well as the anonymous expert reviewers. Their dedicated efforts have been instrumental in ensuring the high quality of the selected submissions, making this collection a valuable resource for the industry.
